# Inbreeding Alters the Gut Microbiota of the Banna Minipig

**DOI:** 10.3390/ani10112125

**Published:** 2020-11-16

**Authors:** Limin Wei, Bo Zeng, Siyuan Zhang, Feng Li, Fanli Kong, Haixia Ran, Hong-Jiang Wei, Jiangchao Zhao, Mingzhou Li, Ying Li

**Affiliations:** 1 Farm Animal Genetic Resources Exploration and Innovation Key Laboratory of Sichuan Province, Sichuan Agricultural University, Chengdu 611130, China; pearl_0329@163.com (L.W.); apollobovey@163.com (B.Z.); siyuan92@163.com (S.Z.); lifeng17512@163.com (F.L.); ranchujiu@163.com (H.R.); 2 Guangdong Provincial Key Laboratory of Animal Molecular Design and Precise Breeding, School of Life Science and Engineering, Foshan University, Foshan 528231, China; 3 College of Life Science, Sichuan Agricultural University, Ya’an 625014, China; fkong@sicau.edu.cn; 4 Key Laboratory of Animal Gene Editing and Animal Cloning in Yunnan Province, Yunnan Agricultural University, Kunming 650201, China; hongjiangwei@126.com; 5 Department of Animal Science, Division of Agriculture, University of Arkansas, Fayetteville, AR 72701, USA; jzhao77@uark.edu

**Keywords:** gut microbiota, inbreeding, Diannan small-ear pig, Banna minipig inbred, 16S rRNA, PICRUSt2

## Abstract

**Simple Summary:**

The mammalian gut microbiota is an indispensable part of host health. The gut microbiota plays a crucial role in nutrient digestibility, preventing colonization of pathogens and maintaining the host immune system. Host genetics has been conclusively shown to closely related to gut microbiota. Inbreeding can cause a decrease of the host’s genetic diversity, however, remarkably little is understood about the gut microbiota of pigs during inbreeding. The Banna minipig inbred is the world’s first successful large mammalian experimental animal inbred line since 1980 from full and half-siblings of the Diannan small-ear pig. Now, Banna minipig inbred has been inbred for over 37 generations, and the inbreeding coefficient is more than 99%. This study is the first to characterize and compare the composition and function of gut microbiota between the Diannan small-ear pig and Banna minipig inbred, aiming to better understand the influence of inbreeding on the gut microbiota.

**Abstract:**

The gut microbiota coevolve with the host and can be stably transmitted to the offspring. Host genetics plays a crucial role in the composition and abundance of gut microbiota. Inbreeding can cause a decrease of the host’s genetic diversity and the heterozygosity. In this study, we used 16S rRNA gene sequencing to compare the differences of gut microbiota between the Diannan small-ear pig and Banna minipig inbred, aiming to understand the impact of inbreeding on the gut microbiota. Three dominant bacteria (*Stenotrophlomonas*, *Streptococcus*, and *Lactobacillus*) were steadily enriched in both the Diannan small-ear pig and Banna minipig inbred. After inbreeding, the gut microbiota alpha diversity and some potential probiotics (*Bifidobacterium*, *Tricibacter*, *Ruminocaccae*, *Christensenellaceae*, etc.) were significantly decreased, while the pathogenic *Klebsiella* bacteria was significantly increased. In addition, the predicted metagenomic analysis (PICRUSt2) indicated that several amino acid metabolisms (‘‘Valine, leucine, and isoleucine metabolism’’, ‘‘Phenylalanine, tyrosine, and tryptophan biosynthesis’’, ‘‘Histidine metabolism’’) were also markedly decreased after the inbreeding. Altogether our data reveal that host inbreeding altered the composition and the predicted function of the gut microbiome, which provides some data for the gut microbiota during inbreeding.

## 1. Introduction

The gut microbiota is considered as the second genome of the human body [1]. The gut microbiota in the mammalian gastrointestinal plays a vital role in improving host health [2,3], preventing colonization of pathogens, and maintaining the host immune function [4,5]. As we know, the gut microbiota coevolve with the host and can be stably transmitted to subsequent generations [6]. The vertical transmission of gut microbiota from mother to newborn is thought to cause lifelong host-microbial symbiosis, which plays a crucial part in early infant development [7,8].

Host genetics plays a significant role in the composition and abundance of gut microbiota [9,10,11,12]. The similarity of gut microbiota of closely related species is higher than that of less closely related and unrelated species [13,14,15]. Several studies have evidenced that host genes and variants are associated with the changes in microbial taxa using quantitative trait locus (QTL) or genome-wide association study (GWAS) [11,16,17]. Wang et al. found that the *VDR* gene (encoding vitamin D receptor) was related to comprehensive the variation of gut microbiota and individual taxa [18]. Chen et al. found that many bacterial communities in pig feces and cecal contents are closely related to host candidate genes, which are mainly related to host metabolism, immune function, and signal transduction [13].

As the level of inbreeding increases, the genetic diversity of the host decreases, and the probability of homozygosity of harmful recessive genes increases, which can lead to inbreeding depression [19]. Therefore, it is very difficult to construct inbred lines, because most inbred animals suffer from impaired fertility or lethality [19,20]. The Banna minipig inbred is the first successful large mammalian experimental animal inbred line in the world since 1980, which was inbred by the half-siblings of the Diannan small-ear pig. The Banna minipig inbred not only has clear genetic background, but also has little individual difference [21,22]. Now, Banna minipig inbred has been inbred for over 37 generations, and the inbreeding coefficient is more than 99%. Therefore, it provides an ideal model animal for the study of the effect of inbreeding on the gut microbiota of pigs.

However, there has been little study about the gut microbiota of pigs during inbreeding. Therefore, this article is the first to characterize and compare the composition and function of gut microbiota between the Diannan small-ear pig and Banna minipig inbred, aiming to better understand the effect of inbreeding on the gut microbiota.

## 2. Materials and Methods

### 2.1. Ethics Statement

All animal experiments were approved by the Institutional Animal Care and Use Committee of the Sichuan Agricultural University, Sichuan, China (DKY-2018102014).

### 2.2. Sampling, DNA Extraction, and High-Throughput Sequencing

**Figure 1:**
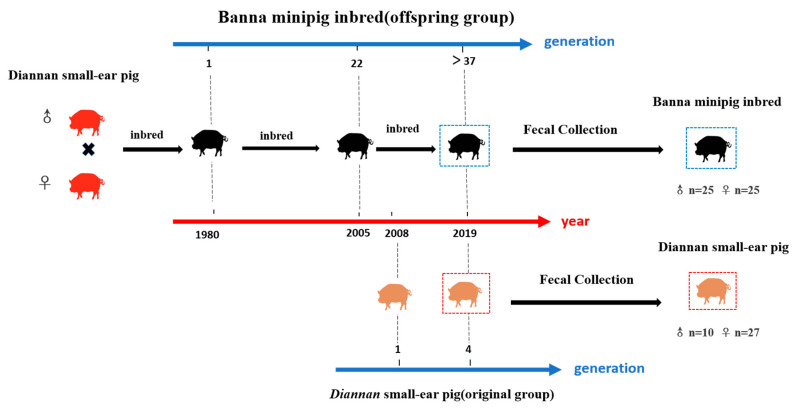
Sample collection of Diannan small-ear pig and Banna minipig inbred. Banna minipig inbred was the inbreeding from full and half-siblings of Diannan small-ear pig in 1980 and now it has been inbred for over 37 generations. The Diannan small-ear pig in this experiment has been conserved for 4 generations since 2008.

The fecal samples of Diannan small-ear pig (n = 37) and Banna minipig inbred (n = 50) were collected from Banna inbred pig farm, Xishuangbanna, Yunnan, China (Figure 1). The diet composition of the Diannan small-ear pig and Banna minipig inbred was similar (corn-soybean based commercial formula diet), and the chemical nutrient composition was shown in Table S1. All pigs in this experiment were healthy and did not use any antibiotics. All fecal samples in experiments were immediately frozen in liquid nitrogen containers and then stored at −80 °C.

According to the manufacturer’s protocol, total bacterial DNA was extracted using TIANamp Stool DNA kit (TIANGEN Biotech, Beijing, China). Bacterial DNA was amplified using the 341F/806R primer pair (341F: CCTAYGGGRBGCASCAG and 806R: GGACTACNNGGGTATCTAAT), targeting the V3-V4 hypervariable region of the bacterial 16S rRNA gene. Then, the PCR was performed using FastStart High Fidelity Enzyme Blend and Phusion^®^ High-Fidelity PCR Master Mix (New England Biolabs). The PCR: 98 °C for 30 s, and 30 cycles of 94 °C for 45 s, and followed by 50 °C for 60 s, 72 °C for 90 s. Check the PCR products in agarose gel electrophoresis, and purify them with the kit (Thermo Fisher Scientific, Wilmington, DE, USA). Ion Plus Fragment Library Kit 48 rxns (Thermo Fisher Scientific, Wilmington, DE, USA) was used to generate the sequencing library. Sequence pooled amplicons was performed by Ion S5TMXL (Thermo Fisher Scientific, Wilmington, DE, USA). 16S rRNA gene amplicons were sequenced at the Novogene Bioinformatics Technology, Co., Ltd. (Beijing, China). The sequencing data were submitted to the National Genomics Data Center (https://bigd.big.ac.cn) (Accession NO. CRA002672).

### 2.3. Sequence Processing and Statistical Analysis

The 16S rRNA gene high-throughput sequencing was analyzed using the QIIME 2 software package (v.2019.4). The amplicon sequence variants were denoised by DADA 2. The PCR primers were trimmed, and the filtered sequences were then dereplicated by DADA 2 function derepFastq to generate unique sequences. Chimeric sequences were removed, and the reads were assigned to operational taxonomic units (OTUs) using de novo OTU picking protocol with a 97% similarity threshold. The Silva database (silva_132_release) was used to annotate taxonomy assignment of OTUs. The alpha diversity analysis of the Shannon index, evenness index, Faith index, and observed OTUs were visualized using GraphPadPrism_v8.0.1. Beta diversity was indicated based on the Bray-Curtis, Jaccard, unweighted, and weighted UniFrac distances. These distances were visualized by principal coordinate analysis (PCoA). We use the phylogenetic investigation of communities by reconstruction of unobserved states (PICRUSt2) to predict the functional profiles of microbial communities [23]. The prediction principle of the predicted metagenomic analysis (PICRUSt2) was as follows. Firstly, the 16S rRNA sequencing results were compared with the Greengenes database, and selected as “closed reference” database has high similarity (default is 97%) OTUs. Secondly, according to the copy number information of the 16S rRNA in the OTU corresponding genome, the number of sequences corresponding to each OTU was divided by its 16S copy number for normalization. Finally, the normalized data were multiplied by the gene content in their corresponding genomes to achieve the purpose of metagenome prediction. The predicted results obtained can be used to classify gene families by KEGG Orthology.

The Mann–Whitney test and analysis of similarities (ANOSIM) were used for the significance test of alpha and beta diversities, respectively. Linear discriminant analysis (LDA) effect size (LEfSe) (LDA score > 2) was used to identify the bacterial taxa of Diannan small-ear pig and Banna minipig inbred at the genus level. The abundance of these features was visualized on a heatmap.

## 3. Results

### 3.1. Taxonomic Classification of the Bacteria Using 16S rRNA Genes

**Figure 2:**
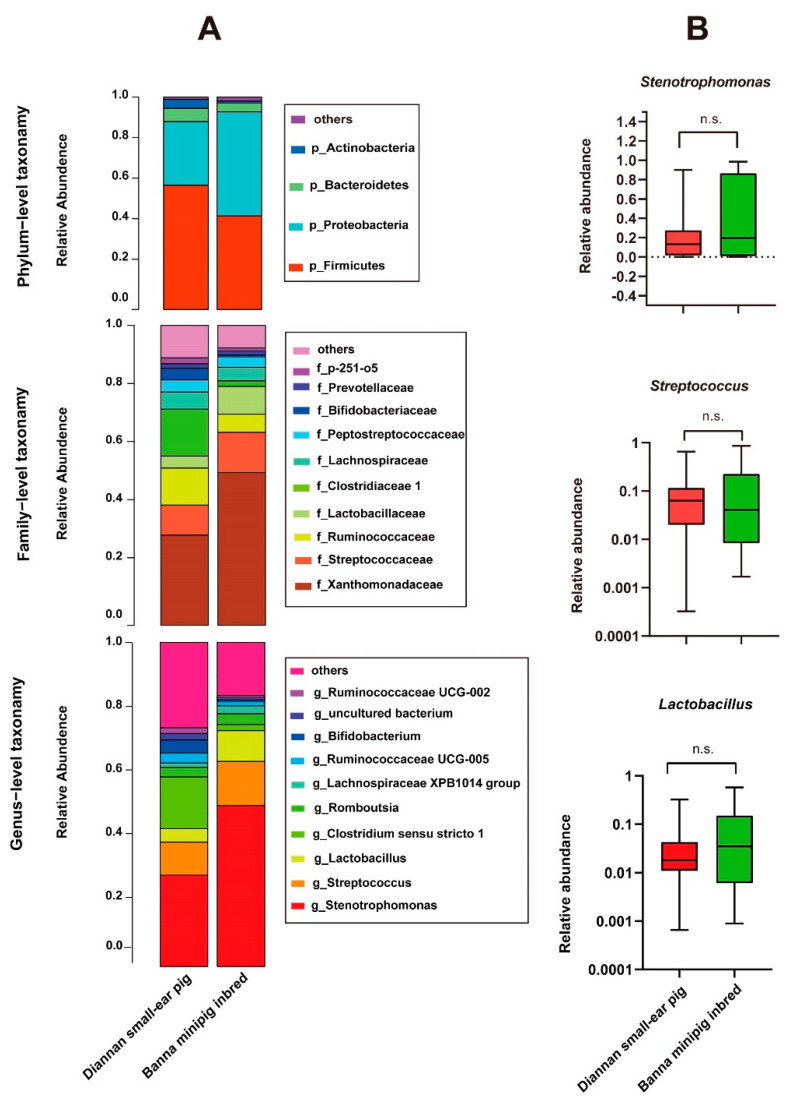
(**A**) The phylum, family, and genus classification of the 16S rRNA gene sequences in Diannan small-ear pig and Banna minipig inbred. (**B**) Distributions of relative abundances are shown as box plots for gut microbiota that was no significant changes in the two groups. n.s., no significance, Mann–Whitney U-test.

We identified a total of 2,983,755 high-quality reads, with an average of 37,168 reads per sample, which ranged from 13,971 to 49,714 (Table S2). In addition, we obtained 8259 operational taxonomic units (OTUs). Figure 2A showed the comparison of relative abundance of fecal microbial composition at phylum, family, and genus levels between Diannan small-ear pig and Banna minipig inbred.

At the phylum level, *Firmicute*, *Proteobacteria*, *Bacteroidetes*, and *Actinobacteria* were predominant in both Diannan small-ear pig and Banna minipig inbred. Around 82.7–97.9% of bacteria in each sample belonged to *Firmicute* and *Proteobacteria*. After inbreeding, the populations of the phylum *Actinobacteria* were remarkably decreased from an average of 4.38% in Diannan small-ear pig to 0.811% in Banna minipig inbred (Whitney test, *p* < 0.01).

At the family level, the three most abundant gut microbiota in Diannan small-ear pigs were mainly composed of *Xanthomonadaceae* (27.81%), *Streptococcaceae* (10.32%), and *Ruminococcaceae* (12.71%). After inbreeding, the relative abundance of *Ruminococcaceae* significantly decreased by 6.44% (Mann–Whitney test, *p* < 0.01).

At the genus level, the top 10 taxa accounted for about 73.5–83.5% of the total readings. The *Stenotrophomonas*, *Streptococcus*, and *Lactobacillus* in both groups were not significantly change during inbreeding (Figure 2B).

### 3.2. Alpha and Beta Diversities

**Figure 3:**
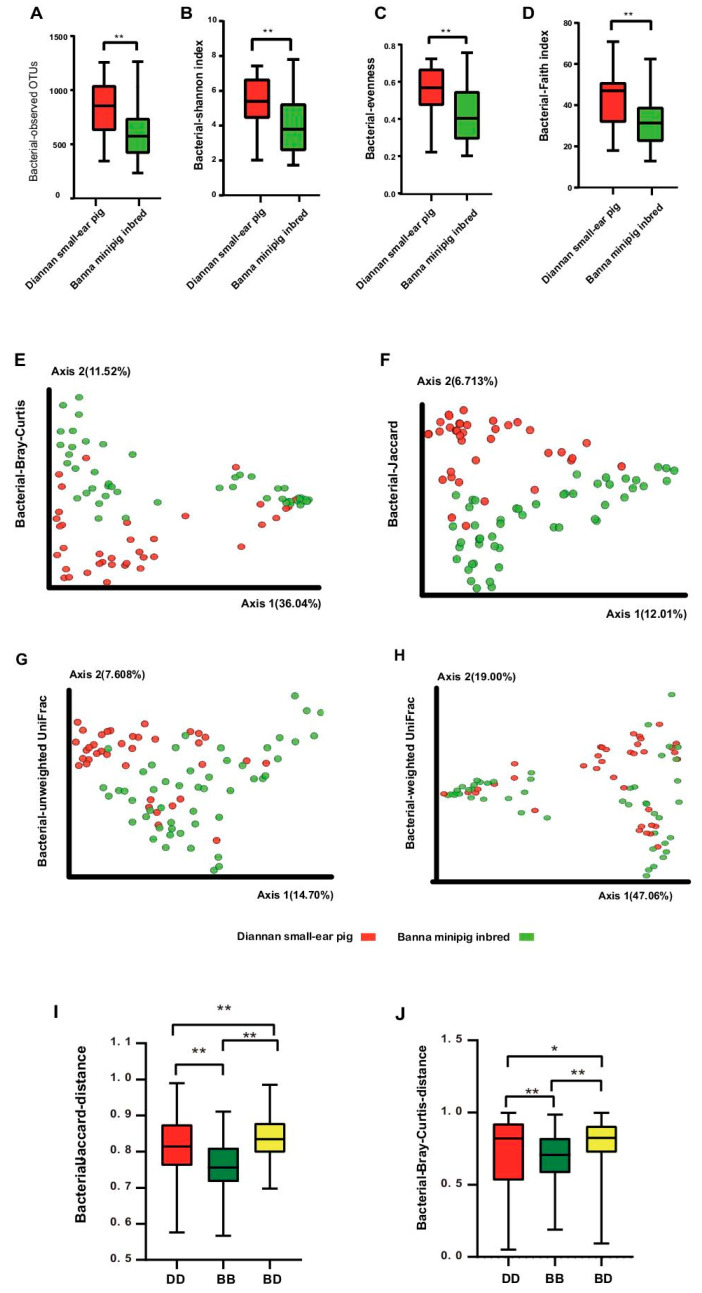
Comparison of alpha and beta diversity of the gut microbiota of Diannan small-ear pig and Banna minipig inbred. Four metrics were used for comparison including the number of observed OTUs (**A**), Shannon index (**B**), evenness index (**C**), and Faith index (**D**) in Diannan small-ear pig and Banna minipig inbred. Red and green bars represent the Diannan small-ear pig and Banna minipig inbred, respectively. ** *p* < 0.01, Mann–Whitney U-test. Jackknifed beta diversity analysis (Figure 3E–J) of gut microbiota for Diannan small-ear pig and Banna minipig inbred. (**E**): PCoA of bacterial Bray–Curtis. (**F**): PCoA of bacterial Jaccard. (**G**): PCoA of bacterial unweighted UniFrac. (**H**): PCoA of bacterial weighted UniFrac. ANOSIM, *p* < 0.01, Red squares and green circles respectively represented Diannan small-ear pig and Banna minipig inbred bacterial communities. Figure 3I,J shows the between-group and within-group distance based on Jaccard (**I**) and Bray–Curtis (**J**). DD: Distance within group of Diannan small-ear pig, BB: Distance within group of Banna minipig inbred, BD: The distance between the Diannan small-ear pig and the Banna minipig inbred. * *p* < 0.05, ** *p* < 0.01, Mann–Whitney U-test.

Four alpha diversity measures, including the Shannon index, evenness index, Faith index, and observed OTUs were calculated. All indices were remarkably higher in the Diannan small-ear pig than those in the Banna minipig inbred (Figure 3A–D, Mann–Whitney test, *p* < 0.001, Table S3). Beta diversity was assessed using PCoA plots based on the Bray–Curtis, Jaccard, unweighted UniFrac and weighted UniFrac distance metrices (Figure 3E–H). The PCoA plot showed that the fecal microbiotas of the Diannan small-ear pig were significantly different from those of the Banna minipig inbred (Table S3, analysis of similarities (ANOSIM), *p* < 0.01). Consistently, the within-group distance was significantly smaller than the between-group distances (Figure 3I,J, Mann–Whitney test, *p* < 0.05, Table S3).

### 3.3. Differences in Bacterial Communities between Diannan Small-Ear Pig and Banna Minipig Inbred

**Figure 4:**
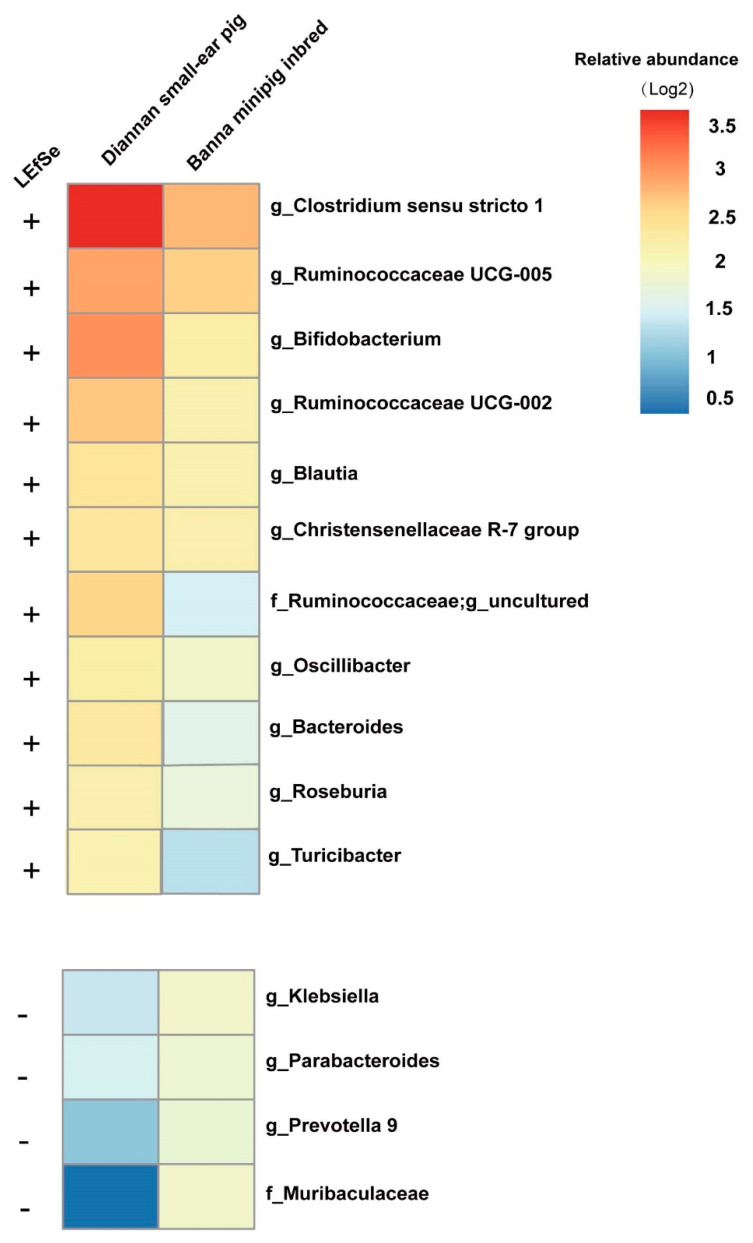
Gut bacterial taxa found to be significantly associated with Diannan small-ear pig and Banna minipig inbred. The average relative abundance of the LEfSe-identified taxa in the Diannan small-ear pig and Banna minipig inbred is plotted as a heatmap. “+” indicates significantly high abundance in Diannan small-ear pig, “−” significantly high abundance in Banna minipig inbred (*p <* 0.05, LDA cutoff = 2.0).

LEfSe analysis was conducted based on genera level at a relative abundance of at least 0.1% to identify specific bacterial taxa in both the Diannan small-ear pig and Banna minipig inbred (Figure 4). Compared with Diannan small ear pigs, 11 taxa (e.g., *Clostridium sensu stricto1*, *Ruminococcaceae UCG-005*, *Bifidobacterium*, *Christensenellaceae R-7 group, Blautia*, *Roseburia,* and *Turicibacter*, etc. *p* < 0.05, LDA cutoff = 2.0) were significantly decreased in the Banna minipig inbred, while 4 taxa (e.g., *Klebsiella*, *Parabacteroides*, etc. *p* < 0.05, LDA cutoff = 2.0) were significantly increased.

### 3.4. The Predicted Function of Gut Microbiota in Diannan Small-Ear Pig and Banna Minipig Inbred

We obtained a total of 1214 closed references and normalized them with 16S rRNA copy number.

Then, used the Kyoto Encyclopedia of Genes and Genomes (KEGG) to predict their metagenomic contributions, and used LEfse to compare the differences KO and pathway between the two groups for further analysis.

**Figure 5:**
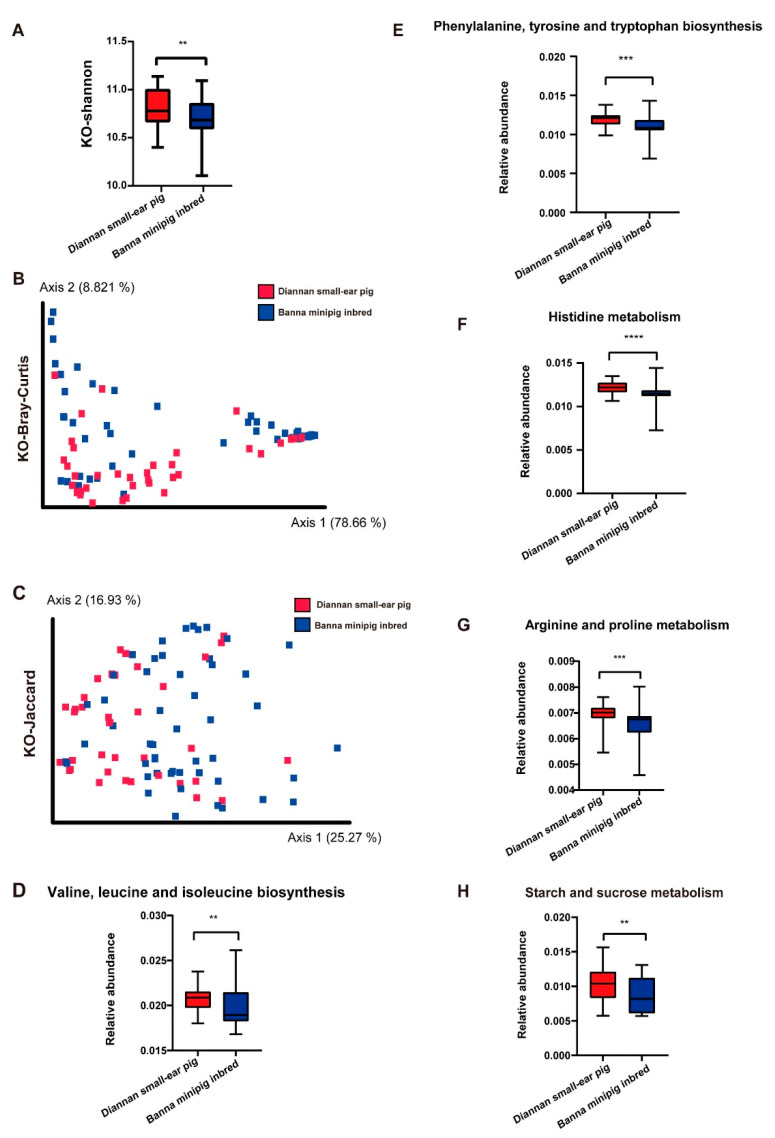
The predicted function of gut microbiota between Diannan small-ear pig and Banna minipig inbred. (**A**): KO–Shannon index between Diannan small-ear pig and Banna minipig inbred. Red and blue bars respectively represent the Diannan small-ear pig and Banna minipig inbred. (**B**): Principle coordinate analysis (PCoA) of KEGG orthologs based on Bray–Curtis distances. (**C**): Principle coordinate analysis (PCoA) of KEGG orthologs based on Jaccard. Red squares and blue circles respectively represent Diannan small-ear pig and Banna minipig inbred. The distance between symbols in the figure reflects the relative difference of community members or structures. The relative abundance of valine, leucine, and isoleucine biosynthesis (**D**), phenylalanine, tyrosine, and tryptophan biosynthesis (**E**), histidine metabolism (**F**), arginine and proline metabolism (**G**), and starch and sucrose metabolism (**H**). Boxplot indicates the interquartile range (IQR). The line in the box represents the middle value, and the error bars respectively represented the lowest and highest values. Mann–Whitney U-test, ** *p* < 0.01, *** *p* < 0.001, **** *p* < 0.0001.

Similar to alpha diversity of the gut microbiota, compared with Diannan small-ear pig, the alpha diversity of KEGG orthologs (KOs) of Banna minipig inbred was significantly reduced. (Figure 5A, *p* < 0.001, Mann–Whitney test). The KOs results showed that 110 KOs were significantly abundant in the Diannan small-ear pig, while only 17 KOs were overrepresented in the Banna minipig inbred (Table S4, *p* < 0.05).

The beta diversity of KOs was assessed using the Bray–Curtis distance metrics and Jaccard and visualized using PCoA (Figure 5B,C). Similarly, in beta diversity observations, the KOs of the Diannan small-ear pig were significantly distinct from those of the Banna minipig inbred (ANOSIM, *p* < 0.01).

The relative abundance of KEGG pathways identified by LEfSe revealed that valine, leucine, and isoleucine metabolism (Figure 5D), phenylalanine, tyrosine, and tryptophan biosynthesis (Figure 5E), histidine metabolism (Figure 5F), arginine and proline metabolism (Figure 5G), and starch and sucrose metabolism (Figure 5H) were significantly more abundant in the Diannan small-ear pig (Mann–Whitney test, *p* < 0.01).

## 4. Discussion

Inbreeding can lead to decrease of host’s genetic diversity, however, little is known about the gut microbiota of pigs during inbreeding. It was reported that in the experiment of 11 generations of inbred mice, the main mode of transmission of the gut microbiota was the vertical transmission. The gut microbiota compositions of the offspring of inbred mice were more similar to that of their ancestors and could be stably transmitted until the 10th generation [24]. A similar phenomenon was also found in our study. In the process of inbreeding, there was no significant change in the three dominant bacteria (*Stenotrophlomonas*, *Streptococcus*, and *Lactobacillus*), which belonged to the TOP 4 of the Diannan small-ear pig and the TOP 3 of the Banna minipig inbred, respectively. *Streptococcus* is generally regarded as a health-promoting microorganism, which can promote human health and the utilization of amino acids in animals [25,26,27,28,29]. *Lactobacillus* can improve feed conversion efficiency in animals [30] and inhibit microorganisms harmful to host health [28]. These abundant bacterial taxa may continuously affect the next generation through vertical transmission. Up to now, the Banna minipig has been inbred for more than 37 generations. Although the ancestor of the Diannan small ear pig in 1980 cannot be found, compared with the current conservation of the Diannan small ear pig, we can still find that these three dominant bacteria are highly conservative and they may have coevolved with the host. These dominant bacterial may be related to maintaining host special traits and promoting host health and immunity.

However, it is well known that inbreeding can cause a decrease of the host’s genetic diversity. It is reported that the Diannan small-eared pigs have high genetic diversity, while the Banna minipig inbred has high homozygosity and a high inbreeding degree [31,32]. Similar to genetic diversity, in our experiments, we also found that the diversity of gut microbiota in Banna minipig inbred was also remarkably decreased. It suggests that the gut microbiota diversity may have a positive correlation with host gene diversity, which is similar to the result reported about Salmon previously [33]. At present, several studies have shown that the gut microbiota diversity plays a vital role in host health. The decrease of microbial diversity may disrupt the normal homeostasis in the body, which has a negative impact on health and leads to disease [34]. Therefore, we speculate that the decrease in gut microbiota diversity may affect the health of the Banna minipig inbred.

Similar to gut microbiota diversity, we also found the abundance of some beneficial bacteria was significantly reduced in the Banna minipig inbred, while the abundance of *Klebsiella* was significantly increased. The abundance of potential probiotics *Bifidobacterium*, *Ruminocaccae*, *Christensenellaceae*, and *Turicibacter* decreased significantly in the Banna minipig inbred. These bacteria can promote host immunity, prevent pathogen attack, and maintain intestinal stability [35,36,37,38]. *Roseburia* and *Blautia* were also decreased in the Banna minipig inbred which are the major bacteria producing butyrate and acetic acid through the fermentation of various substrates [39,40]. Butyrate involves a series of immune responses to prevent inflammation and protect the host against colonic diseases [41,42]. On the other hand, we found a significant increase in lethal *Klebsiella* in the Banna minipig inbred, which can cause early septicemia outbreaks in weaned piglets [43]. The above gut microbiota results indicate that the immune function of the Banna minipig inbred may be reduced, which is consistent with the previous report about the Banna minipig inbred [28]. The reasons for the difference between the Diannan small-ear pig and Banna minipig inbred of gut microbiota are complex. In our experiment, the diet and age of inbred Diannan small-ear pig and Banna minipig inbred were similar.

The PICRUSt2 analysis of the fecal microbial function between the Diannan small-ear pig and Banna minipig inbred gave similar results to the aforementioned analyses. In the process of inbreeding, several amino acid metabolisms were also markedly decreased. The amino acids are considered to be precursors of bacterial synthesis of SCFA (short-chain fatty acids) [44]. It is suggested that there is an interaction between microbial activity and host amino acids and SCFA homeostasis [45]. The metabolism of valine, leucine, and isoleucine were positively correlated with pig feed efficiency [46]. This is because that isobutyric acid is the final product of microbial deamination of valine and is considered to be an indicator of improving protein utilization [47,48]. Previously reported that the biosynthesis of phenylalanine, tyrosine, and tryptophan was related to immune regulation, resistance to inflammation, and regulation of intestinal function [49,50]. Histidine metabolism is closely related to the treatment of metabolic syndrome, inflammatory bowel disease, and nervous system disease [51,52,53]. Arginine and proline pathways are also involved in the regulation of several diseases [54,55], and may become new targets for the treatment of diseases in the future. PICRUSt2 [23] was used to predict the functions of gut microbiota. PICRUSt2 contains an updated database with larger gene family and reference genomes, which can be interoperable with any operational taxon unit (OTU) screening or denoising algorithm. Benchmarking indicates that PICRUSt2 is generally more accurate than PICRUSt and other methods [23]. However, an important limitation of PICRUSt2 and any amplicon-based analysis is that it can only distinguish taxa based on amplified marker gene sequences (e.g., 16S rRNA gene). In addition, the prediction could not distinguish strain-specific functions [23]. Therefore, in the future, we need to use transcriptomics, metabolomics, and fecal transplantation experiments as well as other experiments to further verify the role of these different bacteria in the Banna minipig inbred.

## 5. Conclusions

In this study, we found that three dominant bacteria were steadily enriched in both the Diannan small-ear pig and Banna minipig inbred. These dominant bacteria may play a vital part in sustaining the physiological health and immunity of the host. On the other hand, in the process of inbreeding, with the decrease of host genetic diversity, the diversity of the gut microbiota also showed a similar decline phenomenon, and some potential probiotics bacteria decreased, pathogenic *Klebsiella* increased. In addition, several amino acid metabolisms were also markedly decreased during the inbreeding. Altogether our data reveal that host inbreeding affected the composition and the predicted function of gut microbiome, which provides some data for the gut microbiota during inbreeding.

## Supplementary Materials

The following are available online at https://www.mdpi.com/2076-2615/10/11/2125/s1, Table S1: Chemical nutrient composition, % of DM; Table S2: Sample metadata and OTU table summary; Table S3: The statistical analysis results for Figure 2; Table S4: Differences in Kyoto Encyclopedia of Genes and Genomes (KEGG) orthologs (KO) between Diannan small-ear pig and Banna minipig inbred.
