# Supplementary material for: Inbreeding Alters the Gut Microbiota of the Banna Minipig
Source: Animals (Basel). 2020 Nov 16;10(11):2125. doi: 10.3390/ani10112125 (PMC7697339; doi:10.3390/ani10112125)
Supplement: Supplementary file 1 [file animals-10-02125-s001.pdf]

Table S1. Chemical nutrient composition,% of DM.

Items	Diannan small ear pig	Banna inbred line (female)	Banna inbred line (male)
crude protein≥	12.72	13	15
Crude fiber≤	5.18	9	7
Crude ash≤	5.416	10	9
Lysine≥	0.6502	0.8	0.8

Premix provided the following per kg of dry matter (DM).

Table S2. Sample metadata and OTU table summary.

Sample ID	Groups	breed	Gender	Age (year)	OTUs	Sequence counts
A1	Diannan small-ear pig	Pig	Male	3	20146	68271
A2	Diannan small-ear pig	Pig	Male	3	47549	88439
A3	Diannan small-ear pig	Pig	Male	3	19747	52074
A4	Diannan small-ear pig	Pig	Male	3	20344	60806
A5	Diannan small-ear pig	Pig	Male	3	18339	51560
A6	Diannan small-ear pig	Pig	Male	3	16108	50962
A7	Diannan small-ear pig	Pig	Male	3	49714	85071
A8	Diannan small-ear pig	Pig	Male	3	27218	64188
A9	Diannan small-ear pig	Pig	Male	3	41086	83487
A10	Diannan small-ear pig	Pig	Male	3	26617	69546
A11	Diannan small-ear pig	Pig	Female	4	16691	49703
A12	Diannan small-ear pig	Pig	Female	4	27340	62333
A13	Diannan small-ear pig	Pig	Female	4	37059	86203
A14	Diannan small-ear pig	Pig	Female	4	31921	84645
A15	Diannan small-ear pig	Pig	Female	4	41618	88047
A16	Diannan small-ear pig	Pig	Female	4	17573	53765
A17	Diannan small-ear pig	Pig	Female	4	40181	85457
A18	Diannan small-ear pig	Pig	Female	4	25105	80139
A19	Diannan small-ear pig	Pig	Female	4	41157	84888
A20	Diannan small-ear pig	Pig	Female	4	44334	84016
A21	Diannan small-ear pig	Pig	Female	4	25706	82143
A22	Diannan small-ear pig	Pig	Female	4	23303	67290
A23	Diannan small-ear pig	Pig	Female	4	33568	88673
A24	Diannan small-ear pig	Pig	Female	4	29910	73134
A25	Diannan small-ear pig	Pig	Female	4	27812	53172
A26	Diannan small-ear pig	Pig	Female	4	36911	84229
A27	Diannan small-ear pig	Pig	Female	4	21452	50934
A28	Diannan small-ear pig	Pig	Female	4	14141	50009
A29	Diannan small-ear pig	Pig	Female	4	43470	84894
A30	Diannan small-ear pig	Pig	Female	4	40545	85933
A31	Diannan small-ear pig	Pig	Female	4	30947	71341
A32	Diannan small-ear pig	Pig	Female	4	32115	63917
A33	Diannan small-ear pig	Pig	Female	4	42542	86525
A34	Diannan small-ear pig	Pig	Female	4	21045	56973
A35	Diannan small-ear pig	Pig	Female	4	31218	83159
A36	Diannan small-ear pig	Pig	Female	4	25244	62431
A37	Diannan small-ear pig	Pig	Female	4	22948	54312
X1	Banna minipig inbred	Pig	Female	3	38638	73716
X2	Banna minipig inbred	Pig	Female	3	49435	83976
X3	Banna minipig inbred	Pig	Female	4	26370	51241
X4	Banna minipig inbred	Pig	Female	3	40710	87771
X5	Banna minipig inbred	Pig	Female	4	45943	84045

X6	Banna minipig inbred	Pig	Male	3	26997	53786
X7	Banna minipig inbred	Pig	Female	4	47175	88765
X8	Banna minipig inbred	Pig	Female	3	48623	90123
X9	Banna minipig inbred	Pig	Male	4	40260	88142
X10	Banna minipig inbred	Pig	Female	4	38882	86219
X11	Banna minipig inbred	Pig	Female	4	46478	83161
X12	Banna minipig inbred	Pig	Female	3	40183	82569
X13	Banna minipig inbred	Pig	Female	3	23785	51739
X14	Banna minipig inbred	Pig	Female	4	43741	76143
X15	Banna minipig inbred	Pig	Female	3	33280	82431
X16	Banna minipig inbred	Pig	Male	4	41593	82645
X17	Banna minipig inbred	Pig	Male	4	42932	87447
X18	Banna minipig inbred	Pig	Male	4	49434	89542
X19	Banna minipig inbred	Pig	Male	4	48096	85270
X20	Banna minipig inbred	Pig	Female	4	39550	79363
X21	Banna minipig inbred	Pig	Female	3	36382	83068
X22	Banna minipig inbred	Pig	Female	3	41072	83051
X23	Banna minipig inbred	Pig	Female	4	34286	59061
X24	Banna minipig inbred	Pig	Male	3	39431	88268
X25	Banna minipig inbred	Pig	Male	4	49142	88761
X26	Banna minipig inbred	Pig	Male	4	19287	52264
X27	Banna minipig inbred	Pig	Female	4	43233	85132
X28	Banna minipig inbred	Pig	Female	4	42453	87331
X29	Banna minipig inbred	Pig	Female	4	46818	86998
X30	Banna minipig inbred	Pig	Female	4	21842	63289
X31	Banna minipig inbred	Pig	Male	4	44597	86193
X32	Banna minipig inbred	Pig	Male	3	48598	85257
X33	Banna minipig inbred	Pig	Male	4	22457	57353
X34	Banna minipig inbred	Pig	Female	3	35237	71708
X35	Banna minipig inbred	Pig	Female	4	45702	82578
X36	Banna minipig inbred	Pig	Male	3	42890	86393
X37	Banna minipig inbred	Pig	Female	4	44704	86704
X38	Banna minipig inbred	Pig	Female	4	43618	82207
X39	Banna minipig inbred	Pig	Male	4	47275	85594
X40	Banna minipig inbred	Pig	Male	3	47995	86513
X41	Banna minipig inbred	Pig	Male	4	37168	66270
X42	Banna minipig inbred	Pig	Male	4	22613	77261
X43	Banna minipig inbred	Pig	Male	4	40116	84756
X44	Banna minipig inbred	Pig	Male	3	14298	50509
X45	Banna minipig inbred	Pig	Male	3	13971	51155
X46	Banna minipig inbred	Pig	Male	3	40028	88442
X47	Banna minipig inbred	Pig	Male	3	15104	50406
X48	Banna minipig inbred	Pig	Male	4	38359	88608
X49	Banna minipig inbred	Pig	Male	4	20301	71260
X50	Banna minipig inbred	Pig	Male	3	19949	66926

Table S3. The statistical analysis results for Figure 3.

Fig Number.	Method name	Test used	P-value
Fig 3A	Shannon index	Mann Whitney test	< 0.001
Fig 3B	evenness	Mann Whitney test	< 0.001
Fig 3C	Faith index	Mann Whitney test	< 0.001
Fig 3D	observed OTUs	Mann Whitney test	< 0.001
Fig 3E	Bray-curtis	ANOSIM	< 0.01

Fig 3F	Jaccard	ANOSIM	< 0.01
Fig 3G	unweighted UniFrac	ANOSIM	< 0.01
Fig 3H	weighted UniFrac	ANOSIM	< 0.01
Fig 3I	Jaccard	Mann Whitney test	< 0.01
Fig 3J	Bray-curtis	Mann Whitney test	< 0.05

Table S4. Differences in Kyoto Encyclopedia of Genes and Genomes (KEGG) orthologs (KO) between Diannan small-ear pig and Banna minipig inbred.

Group identified by Linear discriminant analysis (LDA) effect size (LEfSe) with high significance（$P < 0.05$）	KO	KEGG Description
Diannan small-ear pig	K05337	fer; ferredoxin
	K03856	AROA2, aroA; 3-deoxy-7-phosphoheptulonate synthase [EC:2.5.1.54]
	K02598	nirC; nitrite transporter
	K00527	rtpR; ribonucleoside-triphosphate reductase (thioredoxin) [EC:1.17.4.2]
	K06209	pheB; chorismate mutase [EC:5.4.99.5]
	K07402	xdhC; xanthine dehydrogenase accessory factor
	K19294	algI; alginate O-acetyltransferase complex protein AlgI
	K07095	K07095; uncharacterized protein
	K07098	K07098; uncharacterized protein
	K07137	K07137; uncharacterized protein
	K00655	plsC; 1-acyl-sn-glycerol-3-phosphate acyltransferase [EC:2.3.1.51]
	K00873	PK, pyk; pyruvate kinase [EC:2.7.1.40]
	K00876	udk, UCK; uridine kinase [EC:2.7.1.48]
	K04486	E3.1.3.15B; histidinol-phosphatase (PHP family) [EC:3.1.3.15]
	K00850	pfkA, PFK; 6-phosphofructokinase 1 [EC:2.7.1.11]
	K01537	E3.6.3.8; Ca2+-transporting ATPase [EC:3.6.3.8]
	K17320	lplC; putative aldouronate transport system permease protein
	K00615	E2.2.1.1, tktA, tktB; transketolase [EC:2.2.1.1]
	K18475	fliB; lysine-N-methylase [EC:2.1.1.-]
	K16950	asrA; anaerobic sulfite reductase subunit A
	K16951	asrB; anaerobic sulfite reductase subunit B
	K07720	yesN; two-component system, response regulator YesN
	K02004	ABC.CD.P; putative ABC transport system permease protein
	K02003	ABC.CD.A; putative ABC transport system ATP-binding protein
	K05601	hcp; hydroxylamine reductase [EC:1.7.99.1]
	K02026	ABC.MS.P1; multiple sugar transport system permease protein
	K02025	ABC.MS.P; multiple sugar transport system permease protein
	K02029	ABC.PA.P; polar amino acid transport system permease protein
	K10907	K10907; aminotransferase [EC:2.6.1.-]
	K02049	ABC.SN.A; NitT/TauT family transport system ATP-binding protein
	K03310	TC.AGCS; alanine or glycine:cation symporter, AGCS family
	K01448	amiABC; N-acetylmuramoyl-L-alanine amidase [EC:3.5.1.28]
	K01449	cwlJ, sleB; N-acetylmuramoyl-L-alanine amidase [EC:3.5.1.28]
	K07238	TC.ZIP, zupT, ZRT3, ZIP2; zinc transporter, ZIP family
	K06333	cotJB; spore coat protein JB
	K00928	lysC; aspartate kinase [EC:2.7.2.4]
	K03169	topB; DNA topoisomerase III [EC:5.99.1.2]
	K07001	K07001; NTE family protein
	K08600	srtB; sortase B [EC:3.4.22.70]
	K15580	oppA, mppA; oligopeptide transport system substrate-binding protein
	K02760	PTS-Cel-EIIB, celA, chbB; PTS system, cellobiose-specific IIB component [EC:2.7.1.196 2.7.1.205]

K02761	PTS-Cel-EIIC, celB, chbC; PTS system, cellobiose-specific IIC component
K03499	trkA, ktrA; trk system potassium uptake protein
K03498	trkH, trkG, ktrB; trk system potassium uptake protein
K03497	parB, spo0J; chromosome partitioning protein, ParB family
K02810	PTS-Scr-EIIC, scrA, sacP, sacX, ptsS; PTS system, sucrose-specific IIC component
K00700	GBE1, glgB; 1,4-alpha-glucan branching enzyme [EC:2.4.1.18]
K00703	glgA; starch synthase [EC:2.4.1.21]
K01153	hsdR; type I restriction enzyme, R subunit [EC:3.1.21.3]
K10117	msmE; raffinose/stachyose/melibiose transport system substrate-binding protein
K10118	msmF; raffinose/stachyose/melibiose transport system permease protein
K10119	msmG; raffinose/stachyose/melibiose transport system permease protein
K03458	TC.NCS2; nucleobase:cation symporter-2, NCS2 family
K00372	nasA; assimilatory nitrate reductase catalytic subunit [EC:1.7.99.-]
K09936	TC.BAT2; bacterial/archaeal transporter family-2 protein
K03657	uvrD, pcrA; DNA helicase II / ATP-dependent DNA helicase PcrA [EC:3.6.4.12]
K00688	PYG, glgP; glycogen phosphorylase [EC:2.4.1.1]
K01338	lon; ATP-dependent Lon protease [EC:3.4.21.53]
K01489	cdd, CDA; cytidine deaminase [EC:3.5.4.5]
K08303	K08303; putative protease [EC:3.4.-.-]
K06409	spoVB; stage V sporulation protein B
K07335	bmpA, bmpB, tmpC; basic membrane protein A and related proteins
K01992	ABC-2.P; ABC-2 type transport system permease protein
K01193	INV, sacA; beta-fructofuranosidase [EC:3.2.1.26]
K00656	E2.3.1.54, pflD; formate C-acetyltransferase [EC:2.3.1.54]
K04759	feoB; ferrous iron transport protein B
K04758	feoA; ferrous iron transport protein A
K07718	yesM; two-component system, sensor histidine kinase YesM [EC:2.7.13.3]
K13993	HSP20; HSP20 family protein
K06871	K06871; uncharacterized protein
K07736	carD; CarD family transcriptional regulator
K03737	por, nifJ; pyruvate-ferredoxin/flavodoxin oxidoreductase [EC:1.2.7.1 1.2.7.-]
K08384	spoVD; stage V sporulation protein D (sporulation-specific penicillin-binding protein)
K02016	ABC.FEV.S; iron complex transport system substrate-binding protein
K02015	ABC.FEV.P; iron complex transport system permease protein
K02013	ABC.FEV.A; iron complex transport system ATP-binding protein [EC:3.6.3.34]
K17319	lplB; putative aldouronate transport system permease protein
K17318	lplA; putative aldouronate transport system substrate-binding protein
K01286	E3.4.16.4; D-alanyl-D-alanine carboxypeptidase [EC:3.4.16.4]
K08884	serine/threonine protein kinase, bacterial [EC:2.7.11.1]
K02032	ABC.PE.A1; peptide/nickel transport system ATP-binding protein
K02033	ABC.PE.P; peptide/nickel transport system permease protein
K02034	ABC.PE.P1; peptide/nickel transport system permease protein
K02035	ABC.PE.S; peptide/nickel transport system substrate-binding protein
K03518	coxS; aerobic carbon-monoxide dehydrogenase small subunit [EC:1.2.5.3]
K04069	pflA, pflC, pflE; pyruvate formate lyase activating enzyme [EC:1.97.1.4]
K02050	ABC.SN.P; NitT/TauT family transport system permease protein
K02051	ABC.SN.S; NitT/TauT family transport system substrate-binding protein
K02056	ABC.SS.A; simple sugar transport system ATP-binding protein [EC:3.6.3.17]
K02057	ABC.SS.P; simple sugar transport system permease protein
K03892	arsR; ArsR family transcriptional regulator, arsenate/arsenite/antimonite-responsive transcriptional repressor
K03091	sigH; RNA polymerase sporulation-specific sigma factor
K03798	ftsH, hflB; cell division protease FtsH [EC:3.4.24.-]
K03308	TC.NSS; neurotransmitter:Na+ symporter, NSS family
K03111	ssb; single-strand DNA-binding protein
K07053	E3.1.3.97; 3',5'-nucleoside bisphosphate phosphatase [EC:3.1.3.97]

	K03324	yjbB; phosphate:Na+ symporter
	K00266	gltD; glutamate synthase (NADPH/NADH) small chain [EC:1.4.1.13 1.4.1.14]
	K01200	pulA; pullulanase [EC:3.2.1.41]
	K01223	E3.2.1.86B, bglA; 6-phospho-beta-glucosidase [EC:3.2.1.86]
	K02759	PTS-Cel-EIIA, celC, chbA; PTS system, cellobiose-specific IIA component [EC:2.7.1.196 2.7.1.205]
	K02757	PTS-Bgl-EIIC, bglF, bglP; PTS system, beta-glucoside-specific IIC component
	K00936	pdtaS; two-component system, sensor histidine kinase PdtaS [EC:2.7.13.3]
	K03488	licT, bglG; beta-glucoside operon transcriptional antiterminator
	K07240	chrA; chromate transporter
	K09779	K09779; uncharacterized protein
	K00385	asrC; anaerobic sulfite reductase subunit C
	K12257	secDF; SecD/SecF fusion protein
	K00971	manC, cpsB; mannose-1-phosphate guanylyltransferase [EC:2.7.7.13]
	K00975	glgC; glucose-1-phosphate adenylyltransferase [EC:2.7.7.27]
Diannan small-ear pig	K06966	ppnN; pyrimidine/purine-5'-nucleotide nucleosidase [EC:3.2.2.10 3.2.2.-]
	K07304	msrA; peptide-methionine (S)-S-oxide reductase [EC:1.8.4.11]
	K00425	cydA; cytochrome bd ubiquinol oxidase subunit I [EC:1.10.3.14]
	K00426	cydB; cytochrome bd ubiquinol oxidase subunit II [EC:1.10.3.14]
	K03722	dinG; ATP-dependent DNA helicase DinG [EC:3.6.4.12]
	K03293	TC.AAT; amino acid transporter, AAT family
	K03294	TC.APA; basic amino acid/polyamine antiporter, APA family
	K03316	TC.CPA1; monovalent cation:H+ antiporter, CPA1 family
	K01259	pip; proline iminopeptidase [EC:3.4.11.5]
	K01447	xlyAB; N-acetylmuramoyl-L-alanine amidase [EC:3.5.1.28]
	K00059	fabG; 3-oxoacyl-[acyl-carrier protein] reductase [EC:1.1.1.100]
	K01239	iunH; purine nucleosidase [EC:3.2.2.1]
	K01142	E3.1.11.2, xthA; exodeoxyribonuclease III [EC:3.1.11.2]
	K00432	gpx; glutathione peroxidase [EC:1.11.1.9]
	K03305	TC.POT; proton-dependent oligopeptide transporter, POT family
	K03811	pnuC; nicotinamide mononucleotide transporter
	K07386	pepO; putative endopeptidase [EC:3.4.24.-]
